# Recent Advances on Inorganic Nanoparticle-Based Cancer Therapeutic Agents

**DOI:** 10.3390/ijerph13121182

**Published:** 2016-11-25

**Authors:** Fenglin Wang, Chengyao Li, Jing Cheng, Zhiqin Yuan

**Affiliations:** 1 School of Laboratory Medicine and Biotechnology, Southern Medical University, Guangzhou 510515, China; chengyaoli@hotmail.com; 2 Hunan Key Laboratory of Food Safety Science & Technology, Technology Center of Hunan Entry-Exit Inspection and Quarantine Bureau, Hunan Academy of Inspection and Quarantine, Changsha 410004, China; chengjing4323@163.com; 3 State Key Laboratory of Chemical Resource Engineering, Beijing University of Chemical Technology, Beijing 100029, China

**Keywords:** cancer therapeutic agents, gold nanoparticles, magnetic nanoparticles, upconversion nanoparticles, mesoporous silica nanoparticles

## Abstract

Inorganic nanoparticles have been widely investigated as therapeutic agents for cancer treatments in biomedical fields due to their unique physical/chemical properties, versatile synthetic strategies, easy surface functionalization and excellent biocompatibility. This review focuses on the discussion of several types of inorganic nanoparticle-based cancer therapeutic agents, including gold nanoparticles, magnetic nanoparticles, upconversion nanoparticles and mesoporous silica nanoparticles. Several cancer therapy techniques are briefly introduced at the beginning. Emphasis is placed on how these inorganic nanoparticles can provide enhanced therapeutic efficacy in cancer treatment through site-specific accumulation, targeted drug delivery and stimulated drug release, with elaborations on several examples to highlight the respective strategies adopted. Finally, a brief summary and future challenges are included.

## 1. Introduction

Although our knowledge towards cancer has been significantly improved, cancer is still formidable and it is one of the most leading causes of human death [1]. At present, treatments are mainly based on chemotherapy, radiation and surgery. These treatments can prolong the life expectancy of some patients. However, they also come with a great sacrifice to the patients’ life quality due to the severe side effects [2]. In addition, the relapse and multi-drug resistance make the current treatments ineffective toward partial cancers [3]. Thus, developing functional materials and/or facile methods with enhanced therapeutic efficacy and minimal side effects is of significance to overcome these issues.

With the rapid development in nanotechnology, nanoparticles with sizes ranging from 1 to 100 nm at least in one dimension, play important roles in many fields, such as sensing, bioimaging, catalysis and energy, because of their small sizes, high surface-to-volume ratio and unique optical properties [4,5]. For example, gold nanoparticles show wide applications in the detection of small analytes and biomolecules via surface modification [6]. Semiconducting quantum dots display great potential in bioimaging because of their high fluorescence quantum yield and wide emission range [7]. With high surface-to-volume ratio, metallic nanoparticles or metal oxide nanoparticles usually show high catalytic capability toward organic reactions via reducing the active energy of those reactions [8]. In addition, nanoparticles such as organic nanoparticles (e.g., liposomes and dendrimers), inorganic nanoparticles and organic/inorganic hybrid nanoparticles, etc. have also been developed for cancer treatment [5,9,10,11,12,13]. As compared to other nanoparticle formulations, inorganic nanoparticles have the advantages of facile preparation, excellent biocompatibility and wide surface conjugation chemistry. Inorganic nanoparticle-based cancer therapy has been extensively exploited in the last two decades. Various techniques including photodynamic therapy, hyperthermia and drug delivery are commonly used in developing inorganic nanoparticle-based cancer therapy systems [14,15]. These systems suggest that inorganic nanoparticles are ideal for the development of effective and versatile cancer therapy systems.

Despite of the publication of several excellent review articles on nanomaterial-based cancer therapy systems in the past few years [14,16,17], a brief summary of recent progress of cancer therapy is useful for junior researchers to understand the fundamental principles and realize the differences of cancer therapy performance of various inorganic nanoparticles. In this review, cancer therapy techniques of inorganic nanoparticles are briefly introduced at first. Recent advances in inorganic nanoparticles, including gold nanoparticles, magnetic nanoparticles, upconversion nanoparticles, mesoporous silica nanoparticles and multi-functional nanoparticle-based cancer therapy systems are provided to show their in vitro and in vivo applications in cancer treatment. We have to apologize that only a few examples are selected to highlight the potential use of inorganic nanoparticles in cancer therapy in this review article due to the limited length. Finally, a summary and challenges for inorganic nanoparticle-based cancer therapy systems are briefly discussed. 

## 2. Cancer Treatment Techniques

Before we go into details about each type of inorganic nanoparticle-based cancer therapy, the mechanisms of several cancer treatment techniques, including photodynamic therapy, hyperthermia and drug delivery are briefly introduced. In addition, how the introduction of inorganic nanoparticles can bring enhanced treatment efficacy to these techniques is discussed.

### 2.1. Photodynamic Therapy

The first one we are going to introduce is photodynamic therapy (PDT). PDT is a technique that involves light, a photosensitizer and molecular oxygen. When the photosensitizer is irradiated with light of an appropriate wavelength, the photosensitizer becomes excited and can generate reactive oxygen species by interacting with the surrounding molecular oxygen and subsequently kill cancer cells [18]. For instance, Niagara et al. designed an upconversion nanoparticle-based PDT for in vivo tumor growth inhibition in a mouse model [19]. Both merocyanine 540 and zinc phthalocyanine were entrapped within the silica encapsulated upconversion nanoparticles. As the absorption spectra of photosensitizers overlap with the emission spectrum of upconversion nanoparticles, the photosensitizers are activated and cytotoxic singlet oxygen is generated when irradiated with a 980 nm laser. PDT has been introduced for cancer treatment as well as providing antimicrobial and antifungal effects [20].

Although PDT has many advantages such as non-invasiveness, fewer side effects and ease of operation as compared to conventional treatments (i.e., surgery, radiation therapy and chemotherapy), its application in clinics still has some hurdles. One limitation of PDT is the systemic distribution of photosensitizers, which may lead to some undesirable side effects [21]. This could be improved by associating nanoparticles with photosensitizers. Due to the enhanced permeability retention effect, the nanoparticles would have a higher chance of accumulating at the tumor sites [22]. In addition, surface functionalization of nanoparticles with ligands can provide specific targeting [23]. Another limitation of PDT is the limited penetration depth of light, which results in low therapeutic efficiency. As is known, the transparent window of tissue is in the near infrared region, ranging from 700 to 1100 nm [24]. Hence, the therapeutic depth of PDT would be greatly increased if near infrared light is used. However, there are very few photosensitizers that can be excited with near infrared light [25]. This could be overcome with upconversion nanoparticles (UCPs) that can convert near-infrared (NIR) light to ultraviolet or visible light and activate the photosensitizers [26]. Hence, the introduction of nanoparticles can endow PDT with better performance for disease treatment. Additionally, majority of PDT–based cancer treatment generally relies on the photosensitized generation of singlet oxygen which may be affected by tumor hypoxia. More recently, researchers have shown that singlet oxygen can be chemically generated which has the potential to alleviate this problem [27].

### 2.2. Hyperthermia

The second cancer treatment technique is hyperthermia. It has been used in cancer treatment due to heat-induced apoptotic cell death. As cancer cells are more susceptible to heat induced destruction than normal tissue cells, this technique allows surgeons to treat tumors with minimal damage to the surrounding tissue [28]. In addition, hyperthermia can be introduced together with radiation and chemotherapy to enhance their treatment efficacy [15]. For instance, the cytotoxicity of many chemotherapeutic agents increases when the temperature is in the range of 40.5–43 °C [15].

There are different ways to generate hyperthermia. One way is through light irradiation, which is also called photothermal therapy. When an energy source such as a laser is applied to metallic nanoparticles, the nonionizing electromagnetic radiation energy could be converted to heat due to the electron excitation and relaxation within the metallic nanoparticles. The heat can result in temperature increase of the surrounding tissue, which can induce cell death. It is a relatively noninvasive technique with minimal side effects and ease of operation. However, there are two important aspects to consider for the application of photothermal thearapy for tumor treatment in vivo. First, the energy conversion efficiency of photothermal therapy depends on the laser and surface plasmon resonance (SPR) frequency of metallic nanoparticles. In order to have high penetration depth, near infrared excitation is more desirable. The SPR frequencies of gold nanoshells and gold nanorods can be tuned to the near infrared region via changing the core-shell thickness and aspect ratio, respectively, which may potentially increase therapeutic depth [29]. Another aspect that needs to be considered is the tumor specificity of these nanoparticles. Without specificity, the nanoparticles have a systemic distribution which may increase side effects and make it very difficult to heat deep tumors. The specificity of metallic nanoparticle-based photothermal therapy can be greatly improved by modifying their surface with specific ligands [30,31]. Therefore, the introduction of metallic nanoparticles can further improve the performance of photothermal therapy-based cancer treatment. 

Another way to generate heat is by exposing magnetic nanoparticles to an alternating magnetic field [32]. The most common nanoparticles are iron oxide nanoparticles, which are also called superparamagnetic iron oxide nanoparticles (SPIONs). The alternating magnetic field can flip their magnetic polarity and the hysteric loss during flipping is converted into heat, which causes a rise in the local temperature [15]. Magnetic nanoparticle-based hyperthermia has the advantages of noninvasiveness, site-specificity and deep therapeutic depth [33]. However, short circulation and cytotoxicity are the two shortcomings of the magnetic-based hyperthermia [34]. These could be overcome by surface functionalization of the magnetic nanoparticles [35]. 

Besides developing hyperthermia with different types of nanoparticles, researchers have also been trying to control and monitor temperature more precisely. For instance, Zhu et al. developed a carbon-coated core-shell upconversion nanocomposite for photothermal therapy that has temperature-feedback properties. Real-time microscopic temperature monitoring during photothermal therapy was achieved. With this system, photothermal ablation of tumor was achieved in high spatial resolution with minimal damage to normal tissue in vivo [36].

### 2.3. Drug Delivery

Another therapy technique is drug delivery, which delivers the therapeutic agents (drug molecules or genes) via a carrier to certain sites. Many investigations have demonstrated that the distribution profiles of therapeutic agents in tissues and cells can be controlled by entrapping them in the submicronic colloidal systems, which can increase antitumor efficacy and reduce systemic side effects as well [16,17,37].

Similar to other organic-based drug delivery systems such as the liposome and polymer-based ones, the potential applications of inorganic nanoparticles have also been extensively explored for various types of inorganic nanoparticles. As compared to other organic drug delivery systems, inorganic nanoparticles as drug carriers have their advantages. In general, inorganic nanoparticle-based drug delivery systems have more versatile surface modification strategies. Attributed to their easy surface functionalization and unique physico-chemical properties, many different strategies have been introduced to combine therapeutic agents with nanoparticles. For instance, drug molecules can be loaded into nanoparticles via hydrophobic interaction, electrostatic interaction and covalent bonding with certain liable groups which can be cleaved by enzymes or external stimuli to achieve responsive releasing [14,17,38]. Associating therapeutic agents with nanoparticles can provide several advantages. First, the delivery capacity can be increased for certain agents that have poor solubility in physiological conditions. Second, the nanoparticles can also protect the therapeutic agents from degradation. Third, nanoparticles can increase the circulation time and provide a prolonged period of drug release via surface modification. For instance, the surface of the inorganic nanoparticles can be functionalized with molecules such as polyethylene glycol (PEG) to prolong circulation time [37]. Moreover, active targeting can be achieved by incorporating antibodies, ligands, etc. onto the nanoparticle surface [39]. Hence, drug delivery systems based on these strategies can further enhance therapeutic efficacy through site specificity, longer circulation time and active targeting. In addition, various strategies have been incorporated into inorganic nanoparticle-based drug delivery system for the preparation of external stimuli responsive drug release system and drug delivery system with controllable drug release rate [40,41].

## 3. Inorganic Nanoparticle-Based Cancer Therapy

Based on the above techniques, many inorganic nanoparticles have been exploited as potential therapeutic agents in vitro and in vivo. In this section, we will discuss cancer therapy applications using several types of inorganic nanoparticles, including gold nanoparticles, magnetic nanoparticles, upconversion nanoparticles and mesoporous silica nanoparticles. 

### 3.1. Gold Nanoparticles

With the great advance in synthetic strategies, researchers are capable of preparing a variety of gold nanoparticles including nanospheres, nanorods, nanoshells and nanocages [42,43,44,45,46,47]. With proper post-functionalization, gold nanoparticles have been demonstrated to be promising agents for cancer therapy. Gold nanoparticles have been widely investigated as photothermal agents and drug carriers, attributed to their optical properties, biocompatibility, inertness and ease of preparation. The SPR properties of gold nanoparticle make them promising photothermal therapy agents for cancer treatment. Upon the absorption of light, gold nanoparticles can generate heat because of the electron-phonon and phonon-phonon interactions. For instance, localized cell damage was demonstrated with antibody conjugated nanoparticles that are irradiated with shorter laser pulses [48]. In addition, the SPR of gold nanorods, gold nanoshells and gold nanocages can be easily tuned into the near infrared region, which permits deeper tissue penetration and less photodamage.

**Figure 1:**
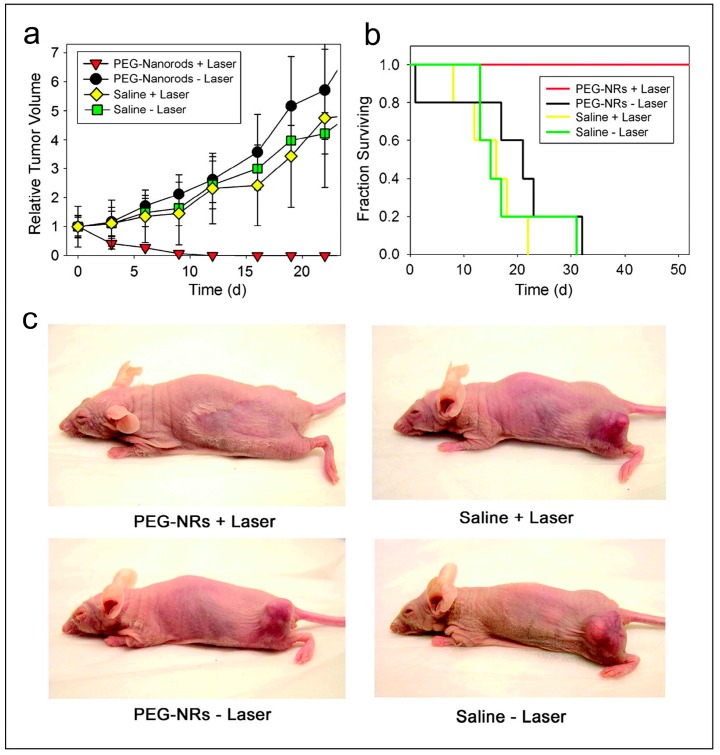
Photothermal destruction of mice bearing MDA-MB-435 human tumors with polyethylene glycol (PEG)-gold-nanorods (PEG-NRs). (**a**) volumetric changes in tumor sizes over time after irradiation. Mice with two human tumors on opposite flanks were injected with either saline or PEG-NRs. The right flank of each mouse was exposed to computationally designed irradiation regimen (810 nm, 2 W/cm^2^, 5 min); (**b**) survival of mice over time after irradiation. Mice with one human tumor were injected with either saline or PEG-NRs and irradiated as in (**a**); (**c**) at 20 days after irradiation, all PEG-NR ejected mice showed no evidence of tumor regrowth while all other treatment groups harbored thriving tumors. Reproduced with permission from ref. [50].

Photothermal therapy with gold nanorods was first reported by Huang et al. High efficacy toward cancer cells was demonstrated in vitro by irradiating the gold nanorods with a continuous near-infrared laser at 800 nm [49]. The possibility of tumor suppression with gold nanorod-based photothermal therapy was demonstrated in vivo using a mice model [50]. The gold nanorods were injected into the tail veins of tumor-bearing mice and exposed to an NIR laser. After 10 days of treatment, the tumors for the ones treated with nanorods completely disappeared when externally observed, while the tumors for the control grow progressively. After 20 days of treatment, there was no tumor regrowth for the ones treated with nanorods, while there was rapid tumor growth for the controls as shown in Figure 1. It was demonstrated that gold nanorods could be used as an effective photothermal agent for tumor suppression in vivo. More recently, Kolemen et al. have investigated endoperoxide-modified gold nanorods as potential photodynamic therapy. Controlled release of singlet oxygen was achieved by plasmonic heating. Chemically generated singlet oxygen induced cancer cell apoptosis was demonstrated in cell culture [27].

The application of gold nanocages as photothermal agents for cancer treatment was also widely explored. For instance, Chen et al. demonstrated the photothermal effect of gold nanocages by destroying breast cancer cells in vitro with anti-epidermal growth factor receptor (anti-EGFR) conjugated gold nanocages [31]. Later, in vivo photothermal efficacy of gold nanocages was studied using a mice tumor model. Based on their results, the tumor-bearing mice intravenously injected with PEGylated gold nanocages were rapidly heated to 50 °C within 1 min and then reached a plateau temperature of 55 °C after 2 min, while there was essentially no temperature change for the controls that were injected with saline [51]. 

Beyond hyperthermia, gold nanoparticles have also been investigated as potential delivery vehicles for drugs and genes. As compared to other organic drug delivery systems such as liposome and polymer-based ones, gold nanoparticle-based drug delivery systems can be more easily functionalized to achieve site-specific delivery and remote-controlled drug release [27]. For instance, gold nanoparticles functionalized with a hydrophobic inner shell and a hydrophilic outer shell were introduced for targeted anticancer drug delivery [52]. In this work, the anticancer drug (doxorubicin) was conjugated onto the inner shell via an acid-cleavable hydrazine linkage while the recognition molecule (folate) was conjugated to the outer shell. With such a versatile design, targeted delivery and acid-triggered drug release were achieved using 4T1 cells. In addition, a noncovalent anticancer drug-gold nanospheres conjugated system was also introduced for the delivery of drugs into tumors [53]. Using noncovalent conjugation strategies, the anticancer drug was rapidly released into the tumor areas within 10 min after ejection and reached a plateau from 1 to 6 h after ejection. It was demonstrated that this system displayed high drug delivery and release efficacy, however, its treatment efficacy on cancer cells or tumors was not studied. Furthermore, gold nanospheres conjugated with tumor-necrosis factor also show high delivery efficiency to tumor-bearing mice [54]. It was observed that the nanoconjugates preferentially accumulated in the tumor vasculature and suppressed the tumor mass more effectively as compared to the free tumor-necrosis factor. Additionally, Mirkin et al. have investigated the use of gold nanospheres conjugated oligonucleotides for intracellular gene regulation, controlling protein expression in cells [55]. Tunable gene knockdown in cells was achieved by tailoring the density of DNA bound onto the surface of gold nanospheres.

### 3.2. Magnetic Nanoparticles

Magnetic nanoparticles have been used as magnetic resonance imaging (MRI) contrast agents due to their superparamagnetic characteristics [56]. In addition to MRI, magnetic nanoparticles have also been widely investigated as hyperthermal therapeutic agents and drug delivery agents in the past two decades because of their facile preparation, easy separation and site-specific accumulation ability [33,57,58]. Magnetic nanoparticles with controllable size, composition and shape can be synthesized via co-precipitation, thermal decomposition and hydrothermal reaction [59,60,61,62]. The synthesized magnetic nanoparticles are usually coated with organic molecules such as surfactants, polymers or inorganic shells (i.e., silica and carbon) to improve their stability and facilitate post-functionalization [63].

Magnetic nanoparticles with superparamagnetic properties could be guided by external magnetic field, making it a promising carrier for oriented delivery. With an external magnetic field, the therapeutic molecules functionalized magnetic nanoparticles can be concentrated and retained at the designated site, which enables the delivery of drugs to desired regions. Meanwhile, MRI can be used to monitor and determine their distribution that acts as indicators for dose optimization. On the basis of this principle, researchers have investigated magnetically-guided drug targeting since the late 1970s. For instance, Liu et al. combined focused ultrasound together with the active delivery of magnetic nanoparticles as a synergistic delivery system for chemotherapeutic agents for the treatment of central nervous system disease which was monitored with MRI [64]. The treatment efficacy was first demonstrated in vitro. Low cell toxicity of the therapeutic magnetic nanoparticles was observed when the tumor cells were incubated with the magnetic nanoparticles, while increased cell toxicity was displayed at the site where the magnet was placed. Then, therapeutic magnetic nanoparticles were delivered into tumor-bearing animals by combined focused ultrasound/magnetic targeted treatment. A significant increased magnetic nanoparticles accumulation and increased tumor progression efficacy were demonstrated in vivo by using the synergistic technique of focused ultrasound and magnetic field, and such a finding suggests the possibility to enhance the delivery efficiency of magnetic nanoparticles through combining magnetic interaction and other techniques.

**Figure 2:**
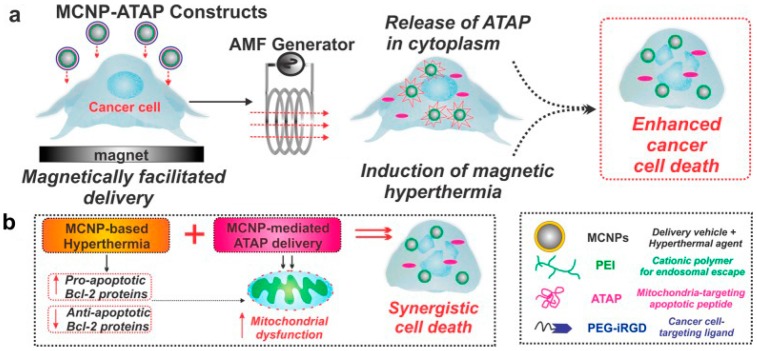
Schematic diagram depicting magnetically targeted delivery of MCNP-ATP to brain and breast cancer cells. (**a**) The release of ATAP from the MCNPs together with the induction of magnetic hyperthermia in the presence of an alternating magnetic field (AMF) results in synergistic cell death; (**b**) MCNP-based hyperthermia can inactivate anti-apoptotic bcl-2 proteins with a concomitant increase in the pro-apoptotic bcl-2 proteins, which results in cancer cell apoptosis, while MCNP-ATP can permeabilize the outer mitochondrial membrane and induce mitochondrial dysfunction. MCNP, magnetic core-shell nanoparticles; PEI, polyethylenimine; ATAP, amphipathic tail-anchoring peptide; PEG, polyethylene glycol; iRGD, internalizing RGD. Reprinted with permission from [65].

Lee et al. have developed magnetic core-shell nanoparticle (MCNP)-based therapeutics [65]. The apoptosis rate of malignant brain and metastatic breast cancer cells is enhanced with a combination of MCNP-mediated delivery and hyperthermia. Figure 2 is the working principle of this platform. The magnetic core was coated with a gold shell, which facilitates the conjugation of an amphipathic tail-anchoring peptide (ATAP) and an internalizing Arg-Gly-Asp (RGD) peptide. The magnetic core is essential to the magnetic guided delivery of the MCNP-ATAP platform as well as the localized hyperthermia. The MCNP-ATAP can permeabilize the outer mitochondrial membrane and cause mitochondrial dysfunction. At the same time, the MCNP-based hyperthermia can inhibit anti-apoptotic bcl-2 proteins and promote pro-apoptotic bcl-2 proteins, which causes apoptosis. This combined therapy shows enhanced cancer cell death via a synergistic interaction mechanism. Enhanced cancer cell apoptosis with ATAP was demonstrated with cancer cells in vitro and the potential of MCNPs to enhance delivery efficacy in tumors was preliminarily demonstrated in vivo with a mouse xenograft model.

### 3.3. Upconversion Nanoparticles

Upconversion nanoparticles (UCPs) possess several unique properties such as super photostability, deep tissue penetration depth and minimal photodamage to biological samples, which make them have wide biological applications, including imaging, detection and therapy [66]. The general synthesis methods for UCPs are co-precipitation, hydrothermal synthesis and thermal decomposition [67,68,69]. Surface modifications such as ligand exchange, chemical conversion of ligands and surface coating are usually performed to improve their stability and dispersity, and facilitate further conjugation [70,71,72]. 

Recently, UCPs have been utilized as drug delivery vehicles as well as photodynamic therapy agents for the development of UCP-based therapy systems. Using doxorubicin as a typical anticancer drug, UCPs have been introduced as drug delivery vehicles through different loading and releasing strategies [73,74,75,76]. For instance, tocopheryl polyethylene glycol 1000 succinate (TPGS) functionalized UCPs were used for doxorubicin delivery [75]. This nanosystem exhibited potent killing ability toward doxorubicin resistant MCF-7 cells as TPGS can inhibit P-glycoprotein expression and facilitate intracellular drug accumulation. 

Several studies report that UCPs are applicable in PDT, as UCPs can be excited by near infrared light and emit UV-visible light, which can activate the photosensitizers. For instance, UCP-based NIR-responsive nanoscale drug delivery systems have been successfully developed. Zhang et al. first reported the NIR (974 nm) light triggered cancer PDT using mesoporous silica coated UCPs loaded with merocyanine-540 [77]. The effective killing of cancer cells was demonstrated in vitro. Later, Liu et al. demonstrated the in vivo treatment of mice tumors based on PDT using UCPs loaded with a photosensitizer and modified with amphiphilic polymers [25]. When the mice were irradiated with a 980 nm laser, singlet oxygen was produced to efficiently destroy the tumors on mice.

**Figure 3:**
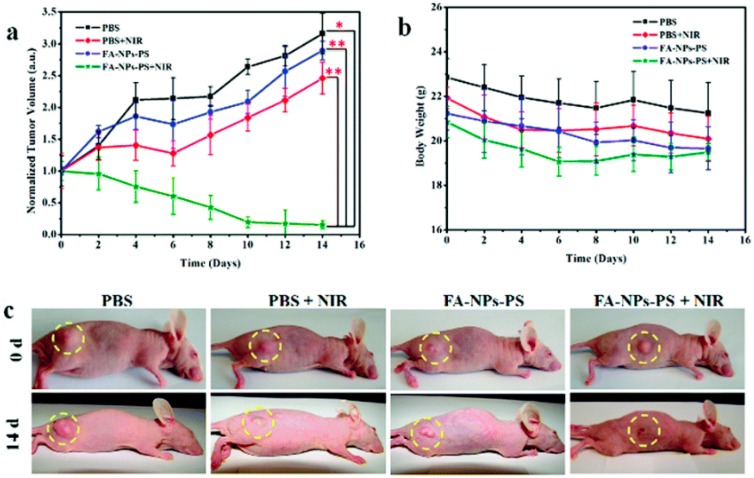
In vivo photodynamic therapy (PDT) characterization of MCF-7 tumor-bearing mice in different groups of phosphate-buffered saline (PBS), PBS + near infrared (NIR), folic acid-functionalized, photosensitizer-loaded Fe_3_O_4_@NaYF_4_: Yb/Er (FA-NPs-PS) and FA-NPs-PS + NIR, (Statistical analysis with Student’s t test: * *p* < 0.05, ** *p* < 0.01). (**a**) The relative tumor volume change in different groups; (**b**) the body weight change of mice in different groups; and (**c**) images of different groups of MCF-7 tumor-bearing mice at the beginning and at the end of 15-day treatments. Reprinted with permission from [78].

Zeng et al. have also demonstrated the NIR PDT with folic acid (FA)-functionalized, photosensitizer (PS)-loaded Fe_3_O_4_@NaYF_4_: Yb/Er (FA-NPs-PS) nanocomposites both in vitro and in vivo [78]. The PDT caused viabilities of MCF-7 and Hela cells were first studied in vitro. The viabilities of MCF-7 and HeLa cells decreased to 18.4% and 30.7% when they were irradiated with NIR for 10 min, while the viabilities for the controls without NIR irradiation were 93.9% and 91.3%, respectively. In addition, the therapeutic efficacy of FA-NPs-PS was investigated in vivo with MCF-7 tumor-bearing nude mice. As shown in Figure 3, the tumor volume in the group of FA-NPs-PS treated with NIR irradiation almost disappeared after 15 days, while the relative tumor size for the control groups significantly increased. These results indicate that MCF-7 tumors could be treated with FA-NPs-PS nanocomposites by NIR-activated PDT. 

### 3.4. Mesoporous Silica Nanoparticles

**Figure 4:**
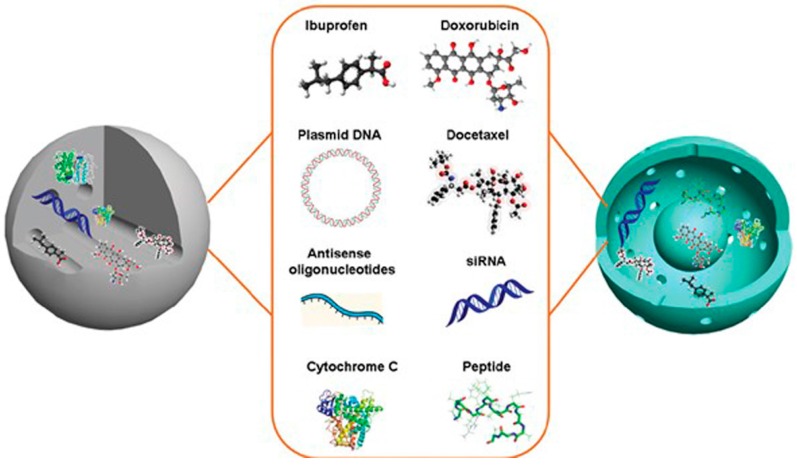
Mesoporous silica nanoparticles as drug delivery systems for various therapeutic agents including drugs (ibuprofen, doxorubicin and docetaxel), therapeutic genes (plasmid DNA, antisense oligonucleotides and siRNA), and therapeutic proteins and peptides. Reprinted with permission from [79].

The tailorable mesoporous structure, high surface area and large pore volume make mesoporous silica nanoparticles excellent drug delivery vehicles [79]. The first mesoporous silica nanoparticles-based drug delivery system was reported in 2001 [80]. Until recently, silica nanoparticles has been used to encapsulate and deliver various drugs such as ibuprofen, doxorubicin, camptothecin, cisplatin, alendronate, peptide drugs, protein drugs and genes, as sketched in Figure 4 [79]. 

**Figure 5:**
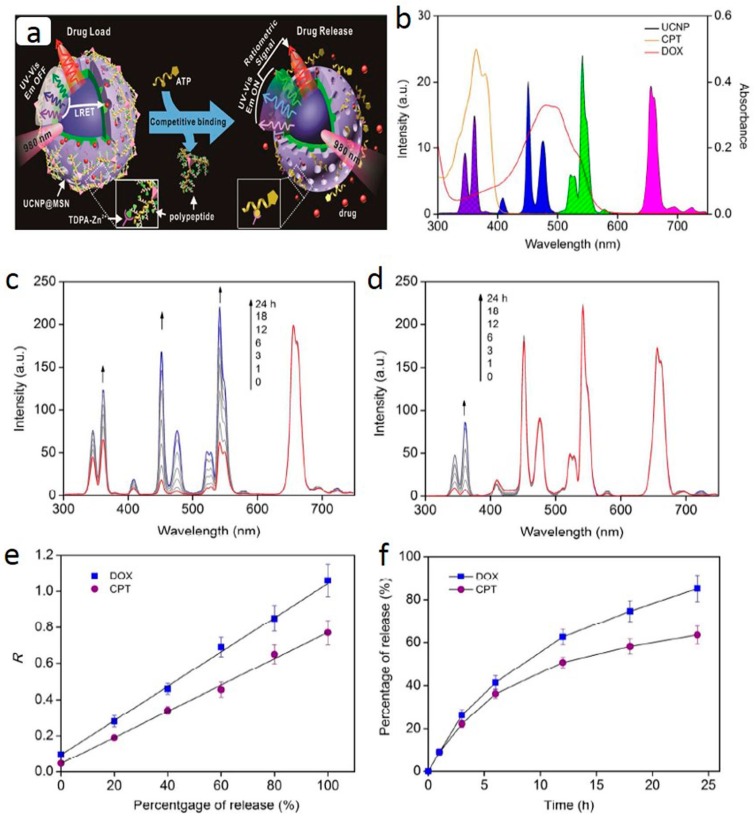
(**a**) schematic drawing of real-time monitoring of ATP-responsive drug release based on polypeptide wrapped zincdipicolylamine-Zn^2+^-upconversion nanoparticles@mesoporous nanoparticles (TDPA-Zn^2+^-UCP@MSN); (**b**) spectrum overlaps between the absorption of anticancer drugs doxorubicin (DOX) and camptothecin (CPT) and the emission of the core-shell UCP@MSN; (**c**,**d**) time-dependent emission spectrum of the polypeptide wrapped TDPA-Zn^2+^-UCP@MSN loaded with DOX and CPT in the presence of 5 mM ATP, respectively; (**e**) linear relationships between the percentage of drug release and the ratiometric signal (R) of the UCP. For DOX, R is the ratio of I_472nm_ to I_656nm_ and R for CPT is the ratio of I_365nm_ to I_656nm_; and (**f**) time-dependent release for DOX and CPT monitored based on the ratiometric emission of UCP. Reproduced with permission from [83].

Moreover, mesoporous silica has also been introduced to coat other nanostructures for drug delivery. For instance, gold nanorods have been coated with mesoporous silica to prepare NIR-responsive nanoscale drug delivery systems. Zhang et al. have introduced a novel therapeutic platform based on mesoporous silica-coated gold nanorods with doxorubicin loading [81]. NIR-light effectively triggers the release of doxorubicin from the loaded nanorods. Meanwhile, there is also a heating effect due to the high NIR-light absorption coefficient of gold nanorods. The synergistic effect of chemotherapy and photothermal therapy provides high therapeutic efficiency in vitro. Later, Shen et al. studied the performance of doxorubicin loaded and mesoporous silica coated gold nanorods in vivo [82]. They found that the tumor growth inhibition effect of this platform was much higher than that of chemotherapy or photothermal therapy alone. More recently, Lai et al. monitored the real-time ATP-stimulated drug release with mesoporous-silica coated multicolor UCPs, as shown in Figure 5 [83]. The mesoporous-silica coated UCPs were functionalized with zinc-dipicolyamine analog on their surface and loaded with polypeptide wrapped chemotherapeutics in mesopores. The presence of ATP could competitively displace the polypeptide and release the entrapped drugs. Real-time drug release was determined by measuring the degree of luminescence energy transfer between the UCPs and entrapped drugs. 

One trend for nanoparticles-based cancer therapy is the integration of multimodal treatment to improve and optimize anticancer efficacy. Magnetic nanoparticles, gold nanoparticles, UCPs and silica nanoparticles can be dedicated to multifunctional construction platforms [84,85]. For example, multifunctions including upconversion imaging, chemotherapy and oxygen sensing were simultaneously achieved by coating the UCPs with a silica layer, and incorporating an anticancer drug and an oxygen sensing molecule into the hydrophobic interspaces between the UCPs and saline [84]. The therapeutic efficacy to cancer cells and cellular oxygen were simultaneously studied. More recently, Ge et al. prepared lanthanide functionalized gold nanoparticles for in vivo imaging and therapy. The nanoparticles were successfully applied to MRI, computed tomography and photothermal therapy for tumor-bearing mice in vivo [86]. Furthermore, hybrid inorganic-organic nanoplatforms have also been introduced for cancer therapy. For instance, by coating mesoporous silica coated gold nanorods with a thermo-responsive polymer shell and a pH-responsive polymer shell, Zhang et al. fabricated polymer encapsulated gold nanorods, which were loaded with doxorubicin. The polymer is both pH sensitive and thermo-responsive. The delivery of heat and anticancer drugs was achieved simultaneously via laser activation mechanisms. With this nanocomposite, tumor growth and lung metastasis were almost completely inhibited via NIR laser irradiation [41]. In addition, Yang et al. developed a light-responsive, singlet-oxygen-triggered drug release platform based on mesoporous silica nanorods for cancer combination therapy. Light-triggered release of both small drugs and larger macromolecules was achieved with high specificity under 660 nm light illumination [40]. 

Another trend for cancer treatment therapy-based nanoparticles is the integration of cancer diagnosis and treatment to one platform, which is called nanotheranostics. For instance, Huang et al. developed a plasmonic gold vesicle for photoacoustic imaging and photothermal therapy. The imaging ability and photothermal therapy efficacy were demonstrated with tumor bearing-mice [87].

## 4. Conclusions

In this review, we have presented a short summary of recent progress of gold nanoparticles, magnetic nanoparticles, upconversion nanoparticles and mesoporous nanoparticles-based in vitro and in vivo cancer therapy systems. Most of these systems show enhanced therapeutic efficiency toward cancer or tumor cells based on PDT, hyperthermia or drug delivery. With versatile design, both in vitro and in vivo therapy is achieved using nanoparticle-based therapeutic platforms. The therapeutic efficacy could be further promoted via a synergistic effect of two or three kinds of therapeutics.

Although inorganic nanoparticle-based cancer therapy has provided numerous advantages for cancer treatment, there are still some concerns and challenges before they can be successfully translocated into clinical applications. First, as cancer therapeutic agents, small size distribution and uniform functionalization are essential for good performance. Hence, it is of great importance to prepare nanoparticle-based therapy with good homogeneity. However, it is still quite challenging to prepare these nanosystems with good reproducibility and homogeneity on a large scale, especially for some complex nanosystems. In addition, the movement and how they function in vivo are very important, especially for drug carrier nanoparticles. For instance, it is still unknown how these nanoparticles penetrate the tumor tissue after deposition [88]. Second, it is quite a challenge to develop nanoparticles with high specificity to the desired sites in vivo, overcoming the physiological barriers such as lung, liver, spleen and kidneys [23]. It is even more difficult to develop nanoplatforms that can bypass the brain blood barriers [89]. Third, another challenge before these nanoplatforms can be really applied in clinical applications is to understand their behavior in vivo, their toxicity, biodistribution and destiny [90]. However, we believe by addressing these issues properly, the translocation of these nanoparticles from laboratory to clinical applications would enter another new era.
